# Elevated non-HDL-C to HDL-C ratio as a marker for NAFLD and liver fibrosis risk: a cross-sectional analysis

**DOI:** 10.3389/fendo.2024.1457589

**Published:** 2024-10-15

**Authors:** Yanyan Xuan, Minghui Zhu, Linzhi Xu, Shujiao Huangfu, Tongyu Li, Chunbo Liu, Dongdong Zhou

**Affiliations:** ^1^ Department of Hospital Infection, The First Affiliated Hospital of Ningbo University, Ningbo, Zhejiang, China; ^2^ Department of Hepatology, The First Affiliated Hospital of Ningbo University, Ningbo, Zhejiang, China; ^3^ Department of General Practice, The First Affiliated Hospital of Ningbo University, Ningbo, Zhejiang, China; ^4^ Department of Hematology, The First Affiliated Hospital of Ningbo University, Ningbo, Zhejiang, China

**Keywords:** NAFLD, non-high-density lipoprotein cholesterol, high-density lipoprotein cholesterol, NHANES, fibrosis

## Abstract

**Background:**

Dyslipidemia is a known independent risk factor for Nonalcoholic fatty liver disease (NAFLD). However, the relationship between NAFLD and the serum non-high-density lipoprotein cholesterol (non-HDL-C) to high-density lipoprotein cholesterol (HDL-C) ratio remains unclear. This study examined the association between the non-HDL-C to HDL-C ratio and NAFLD prevalence, including liver steatosis and fibrosis levels in the population.

**Methods:**

We conducted a cross-sectional study using data from the National Health and Nutrition Examination Survey (NHANES) 2017–2018, including 4798 participants. Liver ultrasound and Transient Elastography (TE) were used to assess fibrosis and steatosis. Adjusted multivariable regression analyses, subgroup analyses based on BMI and sex, and a generalized additive model were employed to investigate the relationship between the non-HDL-C/HDL-C ratio and NAFLD.

**Results:**

Among the 4798 participants, 39.27% (n = 1,884) had NAFLD. Significant positive correlations between non-HDL-C/HDL-C and NAFLD risk were found across all models, with sex-stratified analyses indicating higher risk in men. Liver fibrosis was also associated with non-HDL-C/HDL-C ratios. The Receiver operating characteristic (ROC) analysis shows non-HDL-C/HDL-C as a better predictor for NAFLD than non-HDL-C or HDL-C alone.

**Conclusion:**

Elevated non-HDL-C/HDL-C levels are independently associated with increased NAFLD and liver fibrosis risk in the American population, suggesting its utility in predicting NAFLD and related liver fibrosis.

## 1 Introduction

Nonalcoholic fatty liver disease (NAFLD) is the most common chronic liver disease worldwide. According to cross-sectional research, NAFLD affects over 25% of adults globally; over the next ten years, its prevalence will rise to 56% (1). In the United States, there are currently 37.1% of individuals with NAFLD, and by 2030, there will be 109 million NAFLD patients (2). Owing to bad lifestyle choices, including high-calorie meals, sedentary lives, and insufficient exercise, NAFLD has progressively increased in prevalence (3). The economy and health have suffered dramatically due to this sickness. NAFLD has a substantial correlation with hepatocellular carcinoma, obesity, hypertension, chronic renal disease, gastrointestinal tumors, and other elements of metabolic syndrome (4). According to histology, NAFLD is classified into four different conditions: irreversible cirrhosis, fibrosis, steatohepatitis, and simple fatty liver. Patients with liver fibrosis or Nonalcoholic steatohepatitis (NASH) had greater death rates from all causes and liver-related causes, according to several studies. The most accurate way to diagnose NAFLD is by liver biopsy; nevertheless, because of its numerous serious complications, trustworthy, noninvasive methods are desperately needed to identify and classify patients based on their risk and track the fibrotic process in NAFLD (5).

The cause of NAFLD is hepatic fat buildup brought on by aberrant lipid metabolism (6). There are few studies examining the connection between different types of dyslipidemia and the beginning and development of NAFLD, and the results varied. Finding suitable biomarkers and identifying risk factors associated with NAFLD are essential for disease treatment, early diagnosis, and prognosis. Recent research indicates that because lipid and lipoprotein ratios might show interactions between lipid components, they may be more helpful in predicting the risk of type 2 diabetes or NAFLD than individual lipid readings (7). Previous studies have shown that HDL-C, or high-density lipoprotein cholesterol, has anti-inflammatory properties and antioxidant qualities. It also helps the reverse cholesterol transport pathway to remove dietary cholesterol (8). Lowering HDL-C may lower cholesterol outflow and weaken antioxidant properties, affecting the onset of NAFLD. Non-high-density lipoprotein cholesterol, or non-HDL-C, has also been shown in earlier studies to be a valuable marker of NAFLD (9). According to a recent Swedish study on type 2 diabetes, LDL cholesterol was not the most significant indicator of the risk of cardiovascular disease in those with diabetes, and the non-HDL-C/HDL-C ratio had a more substantial impact on coronary heart disease risk than LDL-C and non-HDL-C (10). The ratio of non-HDL-C to HDL-C, as opposed to a single lipoprotein, is a better indicator of several disorders linked to dyslipidemia and is suggestive of complicated lipid issues (11). These findings imply that by combining non-HDL and HDL cholesterol, it would be feasible to assess the start and development of NAFLD and liver fibrosis.

Using information taken from the years 2017 and 2018 the US National Health and Nutrition Examination Survey (NHANES), we carried out a cross-sectional study to investigate the relationship between NAFLD status, hepatic fibrosis, and non-HDL-C/HDL-C ratio in the general US population as identified by Fibroscan.

## 2 Methods

### 2.1 Study design and population

The NHANES was developed and administered by the National Center for Health Statistics (NCHS) to gather objective data on health problems for adults and children in the US and to address new public health challenges (12). The NHANES was an extensive, ongoing, well-designed cross-sectional survey of American civilians to get estimates of nationally representative food and health indicators. Data were provided in two-year cycles, and the study used a stratified, multistage sample methodology (13). The NHANES data are publicly accessible online to researchers worldwide. All survey respondents undergo a thorough evaluation process involving a home interview and an evaluation at a mobile examination center (MEC), including specialized testing, laboratory work, and a physical examination (14). The Research Ethics Review Board of the NCHS approved the NHANES study procedure, and all members completed a written permission form (15).

**Figure 1:**
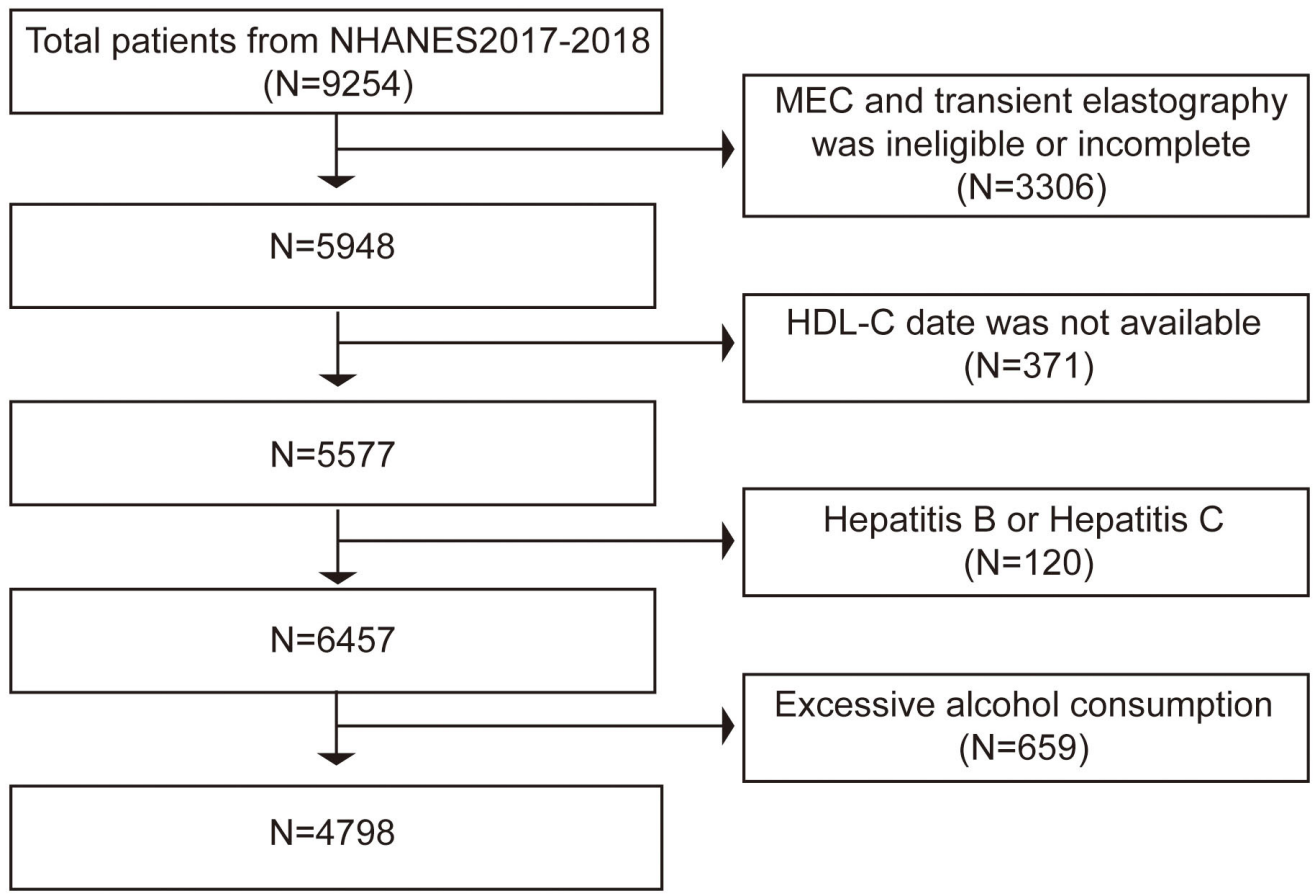
Flowchart of the study participants.

US citizens who participated in the NHANES during 2017 and 2018 made up the study’s participants. The data used in this study comes from the NHANES period from the 2017 to 2018 cycle, which included liver ultrasonography Transient Elastography (TE). In all, 9254 people finished the survey between 2017 and 2018. Of these subjects, 3306 individuals were excluded: those with incomplete tests, those without TE results, and those with preliminary MEC exams. Subsequently, we banned the 371 people for whom HDL-C values were unavailable. Finally, we exclude 50 individuals with hepatitis B (hepatitis B surface antigen-positive) and 70 individuals with hepatitis C (hepatitis C antibody positive) as well as 659 participants who consume excessive amounts of alcohol (defined as males > 21 standard drinks per week and females > 14 standard drinks per week) (16). In the end, the survey comprised 4798 individuals (**Figure 1**).

### 2.2 Definition of NAFLD

NAFLD was assessed using controlled attenuation parameter (CAP) and vibration-controlled transient elastography (VCTE) data. Higher readings indicate increased liver fat content. Based on data from an earlier population-based meta-analysis evaluating the disease’s CAP diagnostic cutoffs. A CAP score of at least 274 dB/m was used to diagnose NAFLD (17, 18).

### 2.3 Vibration controlled transient elastography (VCTE)

A biopsy on the liver remains the gold standard for evaluating hepatic fibrosis and steatosis. However, this technique is expensive, has little repeatability, and has been linked to potential consequences, including bleeding or even death (1: 10,000) (19). In clinical practice, it is presently being gradually superseded by VCTE (20). Doctors frequently use VCTE as a noninvasive method in clinical practice to evaluate NAFLD’s incidence and degree of severity. By measuring liver stiffness measurement (LSM) and CAP, a high degree of accuracy was demonstrated in identifying the presence of both liver fibrosis and steatosis.

According to available clinical data, liver steatosis and liver fibrosis rise with the degree of CAP and LSM values, respectively. Exams were deemed dependable for the current study only if at least 10 LSM results with an interquartile range (IQR) of less than 30% were obtained following a minimum 3-hour fast (21).

CAP with a cutoff of ≥274 dB/m for steatosis is employed in VCTE (17). According to research, LSM values of 8.0, 9.7, and 13.7 kPa are indicative of significant liver fibrosis (F2), advanced liver fibrosis (F3), and cirrhosis (F4), respectively (22).

### 2.4 Variables

The formula of non-HDL-C was total cholesterol (TC) less HDL-C. The non-HDL-C/HDL-C was computed by dividing the non-HDL-C by the HDL-C. The prevalence of NAFLD and liver fibrosis was considered the outcome variable, while non-HDL-C/HDL-C was considered the exposure variable. Confounders that have been shown via prior research and clinical practice were used as covariates in this study. In our investigation, the categories utilized as covariates were sex, race/ethnicity, smoking status, statin use, hypertension status, and diabetes status. The following continuous variables were also included as covariates in our analysis: body mass index (BMI), waist circumference, hip circumference, liver enzymes, serum lipids, fast glucose, fast insulin, serum uric acid (SUA), C-reactive protein (CRP), platelet (PLT), and glycated hemoglobin (HbA1c). Hypertension was defined as (1) Blood pressure of at least 140 mm Hg in the systolic or 90 mm Hg in the diastolic range. (2) Anti-hypertensive drugs are being used at the moment. (3) Hypertension is shown by self-reporting (22). By the American Diabetes Association Criteria, the following parameters were utilized to diagnose T2DM: (1) Self-reported diabetes; (2) Anti-diabetic drug usage; (3) measured fasting plasma glucose (FPG) of 126 mg/dl (7 mmol/L) or above; (4) measured random plasma glucose of ≥ 200 mg/dl (11.1 mmol/L); (5) measured HbA1c of ≥ 6.5% (23). The formula for calculating BMI was weight in kilograms divided by height in square meters. A BMI between 25 and 29.9 kg/m2 was considered overweight, while a BMI over 30 kg/m^2^ was considered obese (24). Furthermore, according to the guidelines provided by the Physical Activity Guidelines, the activity level was also classified as active, moderate, or inactive (25).

### 2.5 Statistical analysis

All statistical analyses were performed using the R language (version 4.1.0, https://www.R-project.org) and the statistical program EmpowerStats (version 4. https://www.empowerstats.com) because of the intricate sample design of the NHANES database. Appropriate weighting was used during data analysis to ensure that the findings represent the US population. If the data for continuous variables were normally distributed, they were expressed using weighted means; otherwise, they were presented as the median value or the IQR. Frequencies and proportions have been used to characterize categorical variables. After non-HDL-C/HDL-C was categorized quarterly, three types of logistic regression models were created to investigate the association between non-HDL-C/HDL-C and NAFLD as well as liver fibrosis: Three sets of adjustments have been made: (1) no covariates have been adjusted; (2) age, sex, and race have been adjusted; and (3) all variables have been corrected.

In addition, to identify groups with significant associations, we stratified gender and BMI subgroups using multivariate logistic regression. Furthermore, smooth curve fits and generalized additive models were used to examine the nonlinear relationship between non-HDL-C/HDL-C and NAFLD. Receiver operating characteristic (ROC) curves were used to evaluate the non-HDL-C/HDL-C’s diagnostic efficacy for NAFLD. The best value of non-HDL-C/HDL-C and other lipid parameters were determined using ROC curves to estimate the population’s risk of NAFLD. A statistically significant result was defined as a *p*< 0.05 was considered statistically significant.

## 3 Results

### 3.1 Demographic and clinical characteristics of the study population

Overall, 4798 participants participated in the study. Of all participants, 1,884 persons (or 39.27%) had a diagnosis of NAFLD. With an average age of 43.35 ± 19.45 years, 54.16% of participants were female, and 45.84% were male. The weighted distributions of the characteristics were shown in **Table 1** based on whether or not they were NAFLD. In addition, those with NAFLD tended to be older, male, Mexican American, and smokers and had higher rates of hypertension, statin use and T2DM. Patients with NAFLD exhibited significantly higher levels of Systolic/Diastolic blood pressure (S/DBP), BMI, waist circumference, hip circumference, LSM, triglycerides (TG), non-HDL-C, TC, low-density lipoprotein cholesterol (LDL-C), alanine aminotransferase (ALT), gamma-glutamyl transpeptidase (GGT), aspartate aminotransferase (AST), fast glucose, fast insulin, HbA1c, PLT, CRP, SUA; conversely, those with NAFLD had significantly lower HDL-C and total bilirubin(TBIL)values (*P* < 0.001 for each). Additionally, compared to the NAFLD subgroup, the non-HDL-C/HDL-C ratio in the non-NAFLD group was much lower (2.36 ± 1.19 vs. 3.13 ± 1.34, *P* < 0.0001). There were, however, no discernible differences in the levels of physical exercise.

**Table 1:** Weighted characteristics of participants with and without NAFLD status.

Characteristics	Non-NAFLD (n = 2914)	NAFLD (n=1884)	*P* value
Age (years)	39.70 ± 19.76	49.11 ± 17.44	< 0.0001
Age (%)			< 0.0001
<20	19.04	4.83	
≥ 20, <40	35.71	26.98	
≥ 40, <60	25.41	37.85	
≥ 60	19.83	30.34	
Gender (%)			< 0.0001
Male	41.93	52.00	
Female	58.07	48.00	
Race (%)			< 0.0001
Mexican American	8.29	13.38	
Other Hispanic	7.56	6.37	
Non-Hispanic White	60.99	60.33	
Non-Hispanic Black	12.04	9.09	
Non-Hispanic Asian	6.33	5.97	
Other Race	4.80	4.87	
Smoking behavior (%)			< 0.0001
Never smoke	71.12	62.80	
Ever smoke	17.71	24.75	
Current smoke	11.17	12.45	
Statin use (%)	14.69	29.37	< 0.0001
Hypertension (%)			< 0.0001
No	74.37	45.04	
Yes	25.63	54.96	
T2DM (%)			< 0.0001
No	93.89	76.82	
Yes	6.11	23.18	
Physical activity level			0.0192
inactive	48.88	48.08	
moderate	8.07	10.55	
active	43.05	41.37	
BMI (Kg/m^2^)	26.10 ± 5.88	33.66 ± 7.19	< 0.0001
BMI (%)			< 0.0001
< 25	47.20	6.28	
≥ 25, <30	32.22	27.84	
≥ 30	20.59	65.88	
Waist circumference (cm)	90.08 ± 14.95	110.40 ± 15.44	< 0.0001
Hip circumference (cm)	101.23 ± 12.11	114.45 ± 14.46	< 0.0001
SBP	117.87 ± 17.40	126.42 ± 17.08	< 0.0001
DBP	69.68 ± 12.04	73.60 ± 12.26	< 0.0001
PLT (10^9^/L)	245.11 ± 59.26	252.20 ± 63.00	< 0.0001
CRP (mg/L)	2.76 ± 6.45	4.72 ± 7.64	< 0.0001
Fast glucose (mmol/L)	5.12 ± 1.04	5.90 ± 2.00	< 0.0001
Fast insulin (mIU/L)	9.41 ± 8.13	17.74 ± 15.10	< 0.0001
Glycohemoglobin (%)	5.42 ± 0.62	5.91 ± 1.04	< 0.0001
ALT (IU/L)	18.33 ± 13.18	26.83 ± 18.86	< 0.0001
GGT (IU/L)	20.84 ± 24.19	33.58 ± 35.68	< 0.0001
AST (IU/L)	20.47 ± 10.57	22.69 ± 12.36	< 0.0001
TBIL (mg/dL)	0.48 ± 0.30	0.45 ± 0.26	0.0003
TG (mg/dL)	109.72 ± 67.52	173.96 ± 129.74	< 0.0001
TC (mg/dL)	180.83 ± 39.30	189.66 ± 40.16	< 0.0001
HDL-C (mg/dL)	56.77 ± 14.39	48.73 ± 13.73	< 0.0001
Non-HDL-C (mg/dL)	124.06 ± 38.32	140.93 ± 40.13	< 0.0001
LDL-C (mg/dL)	104.91 ± 33.48	112.10 ± 36.23	< 0.0001
SUA (mg/dL)	5.02 ± 1.32	5.71 ± 1.41	< 0.0001
Non-HDL-C/HDL-C (%)	2.36 ± 1.19	3.13 ± 1.34	< 0.0001
CAP (dB/m)	215.99 ± 37.14	322.01 ± 35.77	< 0.0001
LSM (kPa)	4.83 ± 2.90	6.95 ± 6.48	< 0.0001

Mean ± SD was for continuous variables. The weighted linear regression model calculated the p-value. % was for categorical variables. The weighted chi-square test calculated the p-value.

NAFLD, Nonalcoholic fatty liver disease; T2DM, Type 2 diabetes; BMI, Body mass index; SBP, Systolic blood pressure; DBP, Diastolic blood pressure; PLT, Platelet; CRP, C-Reactive Protein; ALT, Alanine aminotransferase; AST, Aspartate aminotransferase; GGT, Gamma-glutamyl transpeptidase; TBIL, Total bilirubin; HbA1c, Glycosylated hemoglobin A1c; TG, Triglycerides; TC, Total cholesterol; Non-HDL-C, Non-high-density lipoprotein cholesterol; HDL-C, High-density lipoprotein cholesterol; LDL-C, Low-density lipoprotein cholesterol; SUA, Serum uric acid; LSM, Liver stiffness measurement; CAP, Controlled attenuation parameter.

### 3.2 Correlation between NAFLD and non-HDL-C/HDL-C

The findings of a multiple regression analysis assessing the relationship among non-HDL-C/HDL-C ratios with the risk of NAFLD are shown in **Table 2**. Enormously significant correlations were found in all three multivariable logistic regression models between non-HDL-C/HDL-C and the probabilities of NAFLD: model 1 (OR = 1.787, 95% CI: 1.689, 1.890), model 2 (OR = 1.699, 95% CI: 1.602, 1.801), and model 3 (OR = 1.316, 95% CI: 1.179, 1.469). Furthermore, individuals in Q3 and Q4 had increases in NAFLD risks of 46.2 and 115.4%, respectively, compared to the lowest ratio of non-HDL-C/HDL-C (Q1) in model 3 (*P* for trend < 0.001). The results showed that the risk of developing NAFLD was higher in individuals with higher non-HDL-C/HDL-C ratios than in those with lower ratios.

**Table 2:** Associations between non-high-density lipoprotein cholesterol to high-density lipoprotein cholesterol ratio (non-HDL-C/HDL-C) and NAFLD status.

	Model 1 *OR* (95% CI), *P* value	Model 2 *OR* (95% CI), *P* value	Model 3 *OR* (95% CI), *P* value
**non-HDL-C/HDL-C**	1.787 (1.689, 1.890) < 0.001	1.699 (1.602, 1.801) < 0.001	1.316 (1.179, 1.469) < 0.001
Q1 (0.303-1.282)	Reference	Reference	Reference
Q2 (1.283-2.076)	1.554 (1.288, 1.875) < 0.001	1.508 (1.242, 1.830) < 0.001	1.120 (0.780, 1.610) 0.538
Q3 (2.077-3.204)	3.157 (2.636, 3.781) < 0.001	2.823 (2.343, 3.402) < 0.001	1.462 (1.025, 2.084) 0.035
Q4 (3.205-9.951)	6.500 (5.416, 7.800) < 0.001	5.638 (4.659, 6.822) < 0.001	2.154 (1.495, 3.103) < 0.001
*P* for trend	< 0.001	< 0.001	< 0.001
Subgroup analysis stratified by sex			
**Men**	1.716 (1.588, 1.853) < 0.001	1.672 (1.544, 1.810) < 0.001	1.464 (1.232, 1.740) < 0.001
Q1 (0.303-1.282)	Reference	Reference	Reference
Q2 (1.283-2.076)	1.500 (1.107, 2.031) 0.00889	1.562 (1.139, 2.142) 0.00562	1.049 (0.544, 2.023) 0.886
Q3 (2.077-3.204)	3.272 (2.465, 4.343) < 0.001	3.144 (2.342, 4.220) < 0.001	1.691 (0.906, 3.155) 0.098
Q4 (3.205-9.951)	6.530 (4.962, 8.595) < 0.001	6.189 (4.645, 8.246) < 0.001	3.180 (1.702, 5.940) < 0.001
*P* for trend	< 0.001	< 0.001	< 0.001
**Women**	1.835 (1.686, 1.997) < 0.001	1.731 (1.587, 1.887) < 0.001	1.190 (1.027, 1.380) 0.020
Q1 (0.303-1.282)	Reference	Reference	Reference
Q2 (1.283-2.076)	1.585 (1.248, 2.013) 0.00016	1.487 (1.162, 1.902) 0.00159	1.133 (0.724, 1.773) 0.586
Q3 (2.077-3.204)	3.010 (2.377, 3.811) < 0.001	2.610 (2.047, 3.329) < 0.001	1.268 (0.806, 1.993) 0.304
Q4 (3.205-9.951)	6.132 (4.748, 7.920) < 0.001	5.234 (4.022, 6.813) < 0.001	1.500 (0.929, 2.423) 0.097
*P* for trend	< 0.001	< 0.001	0.085
Subgroup analysis stratified by BMI			
< 25	1.817 (1.561, 2.115) < 0.001	1.485 (1.263, 1.747) < 0.001	1.420 (1.074, 1.878) 0.013
≥ 25, <30	1.477 (1.347, 1.620) < 0.001	1.384 (1.255, 1.527) < 0.001	1.563 (1.288, 1.895) < 0.001
≥ 30	1.354 (1.239, 1.479) < 0.001	1.261 (1.151, 1.382) < 0.001	1.142 (0.970, 1.344) 0.110

Model 1: no covariates were adjusted. Model 2: age, gender, and race were adjusted. Model 3: age, gender, race, hypertension, statin use, waist circumference, hip circumference, BMI, T2DM, smoke, LSM, DBP, SBP, CRP, HbA1c, fast glucose, fast insulin, ALT, AST, GGT, TC, Physical activity, and SUA were adjusted. In the subgroup analysis for gender, the model was not adjusted for gender; in the subgroup analysis for BMI, the model was not adjusted for BMI.

OR Odds ratios, CI confidence interval; other abbreviations are in **Table 1**.

For men, model 1 (OR = 1.716, 95% CI: 1.588, 1.853), model 2 (OR = 1.672, 95% CI: 1.544, 1.810), and model 3 (OR = 1.464, 95% CI: 1.232, 1.740) all demonstrated a strongly positive connection between non-HDL-C/HDL-C and NAFLD risk. For females, we likewise found that all three models: model 1 (OR = 1.835, 95% CI: 1.686, 1.997), model 2 (OR = 1.731, 95% CI: 1.587, 1.88), and model 3 (OR = 1.190, 95% CI: 1.027, 1.380) showed positive correlations. Moreover, in comparison to the lowest ratio of non-HDL-C/HDL-C (Q1) in model 3, male participants in Q4 showed increases in NAFLD risks of 215.4%, which was much higher than females at 50.0%. According to this research, those with greater non-HDL-C/HDL-C levels were likelier to develop NAFLD than those with lower non-HDL-C/HDL-C levels. Furthermore, men were more likely than women to have non-HDL-C/HDL-C elevated levels of NAFLD.

By using a BMI-stratified subgroup analysis, it was shown that those in the BMI group 25-30 kg/m^2^ who had greater non-HDL-C/HDL-C were more likely to develop NAFLD than those in the BMI group < 25 kg/m^2^: model 1 (OR = 1.477, 95% CI: 1.347, 1.620), model 2 (OR = 1.384, 95% CI: 1.255, 1.527), and model 3 (OR = 1.563, 95% CI: 1.288, 1.895).

## 4 Correlation between non-HDL-C/HDL-C and the severity of hepatic steatosis

**Supplementary Table 1** illustrates the relationship between non-HDL-C/HDL-C and the degree of hepatic steatosis based on CAP levels. The P for trend was less than 0.001 in all models: model 1 (β = 16.924, 95% CI: 15.603, 18.245), 2 (β = 14.579, 95% CI: 13.267, 15.891), and 3 (β = 5.638, 95% CI: 3.564, 7.713). The degree of hepatic steatosis increased significantly in the higher non-HDL-C/HDL quartile relative to the lowest quartile (P for trend < 0.001). Furthermore, even after accounting for all confounders, the subgroup analysis stratified by gender showed that there was still a positive connection between the degree of hepatic steatosis and non-HDL-C/HDL-C, and the value was 5.235 (95% CI: 2.428, 8.042, *P* < 0.001) in women and 5.674 (95% CI: 2.475, 8.872, *P* < 0.001) in men.

## 5 Association between non-HDL-C/HDL-C and the severity of liver fibrosis

We also investigated the correlation between the three stages of liver fibrosis and non-HDL-C/HDL-C. Our findings showed that in every model, non-HDL-C/HDL-C was associated with significant liver fibrosis, advanced liver fibrosis, and cirrhosis. After correcting for all covariates, significant liver fibrosis related model 3 (OR = 1.092, 95% CI: 1.009, 1.182), advanced liver fibrosis related model 3 (OR = 1.136, 95% CI: 1.036, 1.247), and cirrhosis related model 3 (OR = 1.142, 95% CI: 1.022, 1.277) were all found associated with non-HDL-C/HDL-C.

Subgroup analysis stratified by gender also reveals that among men, non-HDL-C/HDL-C and the degree of liver fibrosis had positive correlations, according to all three multivariable logistic regression models. Following the adjustment for every covariate, the results for the substantial fibrosis related model 3 (OR = 1.105, 95% CI: 1.001, 1.223), significant fibrosis related model 3 (OR = 1.172, 95% CI: 1.037, 1.323), and cirrhosis related model 3 (OR = 1.207, 95% CI: 1.050, 1.387), were as follows. However, we did not find non-HDL-C/HDL-C linked with the severity of liver fibrosis in females after accounting for all potential confounders in Model 3 (**Table 3**).

**Table 3:** Association between non-HDL-C/HDL-C and risk of hepatic fibrosis.

Degree of hepatic fibrosis	Model 1 *OR* (95% CI), *P* value	Model 2 *OR* (95% CI), *P* value	Model 3 *OR* (95% CI), *P* value
**Significant fibrosis (F2, LSM ≥ 8.0)**	1.209 (1.129, 1.294) < 0.001	1.193 (1.114, 1.278) < 0.001	1.092 (1.009, 1.182) 0.02967
**MEN**	1.171 (1.077, 1.273) < 0.001	1.174 (1.080, 1.277) < 0.001	1.105 (1.001, 1.223) 0.04958
**WOMEN**	1.279 (1.145, 1.430) < 0.001	1.230 (1.096, 1.380) < 0.001	1.058 (0.921, 1.215) 0.42760
**Advanced fibrosis (F3, LSM ≥ 9.7)**	1.215 (1.121, 1.317) < 0.001	1.205 (1.113, 1.305) 0.003	1.136 (1.036, 1.247) 0.00695
**MEN**	1.194 (1.084, 1.315) < 0.001	1.209 (1.099, 1.331) 0.001	1.172 (1.037, 1.323) 0.01078
**WOMEN**	1.260 (1.097, 1.446) 0.00106	1.208 (1.047, 1.393) 0.00952	1.045 (0.881, 1.240) 0.61439
**Cirrhosis** **(F4, LSM ≥ 13.7)**	1.201 (1.084, 1.330) < 0.001	1.203 (1.087, 1.331) < 0.001	1.142 (1.022, 1.277) 0.01915
**MEN**	1.193 (1.063, 1.339) 0.00272	1.221 (1.087, 1.373) < 0.001	1.207 (1.050, 1.387) 0.00795
**WOMEN**	1.227 (0.995, 1.514) 0.05605	1.172 (0.943, 1.456) 0.15327	0.959 (0.742, 1.240) 0.75169

Model 1: no covariates were adjusted. Model 2: age, gender, and race were adjusted. Model 3: age, gender, race, hypertension, BMI, T2DM, smoke, CAP, DBP, SBP, HBA1C, CRP, fast glucose, fast insulin, ALT, AST, GGT, TC, Physical activity, and SUA were adjusted. In the subgroup analysis for gender, the model was not adjusted for gender.

OR Odds ratios, CI confidence interval; other abbreviations are in **Table 1**.

Additionally, we found that the severity of hepatic fibrosis was positively correlated with non-HDL-C/HDL-C based on LSM values in models 1 (β = 0.328, 95% CI: 0.223, 0.434), 2 (β = 0.308, 95% CI: 0.201, 0.415), and 3 (β = 0.223, 95% CI: 0.096, 0.349).In males, all three models still showed a positive relation between non-HDL-C/HDL-C and LSM values in model 1 (β = 0.321, 95% CI: 0.177, 0.466), model 2 (β = 0.301, 95% CI: 0.154, 0.447) and model 3 (β = 0.295, 95% CI: 0.120, 0.471). Nevertheless, this connection was no longer significant in females after controlling for all possible confounders (β = 0.137, 95% CI: -0.054, 0.328), as presented in **Supplementary Table 2**.

## 6 The analysis of the nonlinear relationship

**Figure 2:**
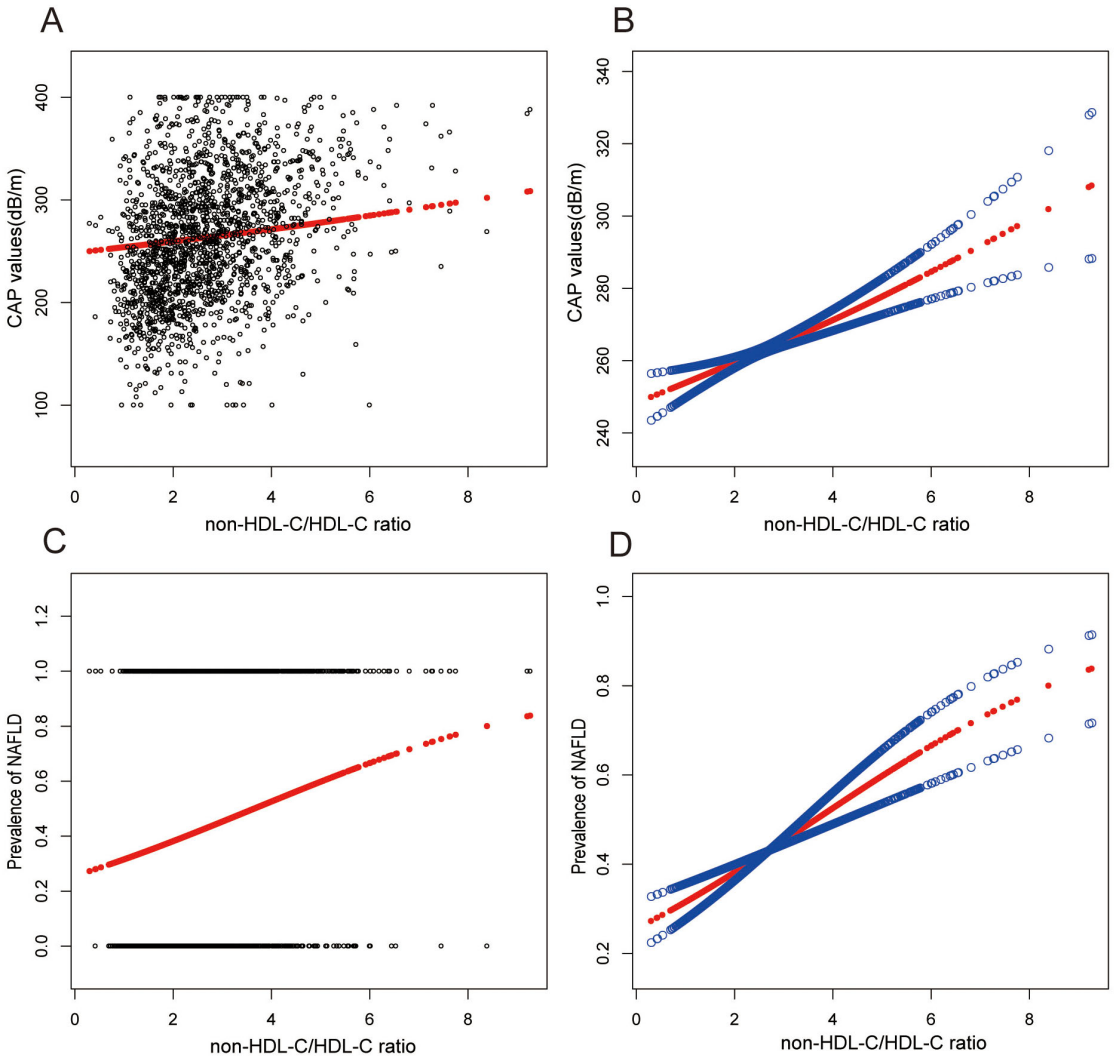
Associations between non-HDL-C to HDL-C ratio and CAP values or prevalence of NAFLD. **(A, B)** Associations between non-HDL-C/HDL-C ratio and CAP values. **(C, D)** Associations between non-HDL-C/HDL-C ratio and prevalence of NAFLD. Each black point represents a sample. The solid red line represents the smooth curve fit between variables. Blue bands represent the 95% confidence interval from the fit. They were adjusted for age, gender, race, hypertension, statin use, waist circumference, hip circumference, BMI, T2DM, smoke, CAP, DBP, SBP, HBA1C, CRP, fast glucose, fast insulin, HbA1c, ALT, AST, GGT, TC, Physical activity, and SUA.

Smooth curve fitting techniques and generalized additive models have delineated the nonlinear relationship between non-HDL-C/HDL-C and NAFLD. It was found that non-HDL-C/HDL-C exhibited a positive association with CAP values and the prevalence of NAFLD, as depicted in **Figure 2**.

## 7 Non-HDL-C/HDL-C as a predictor of NAFLD: ROC analysis

**Figure 3:**
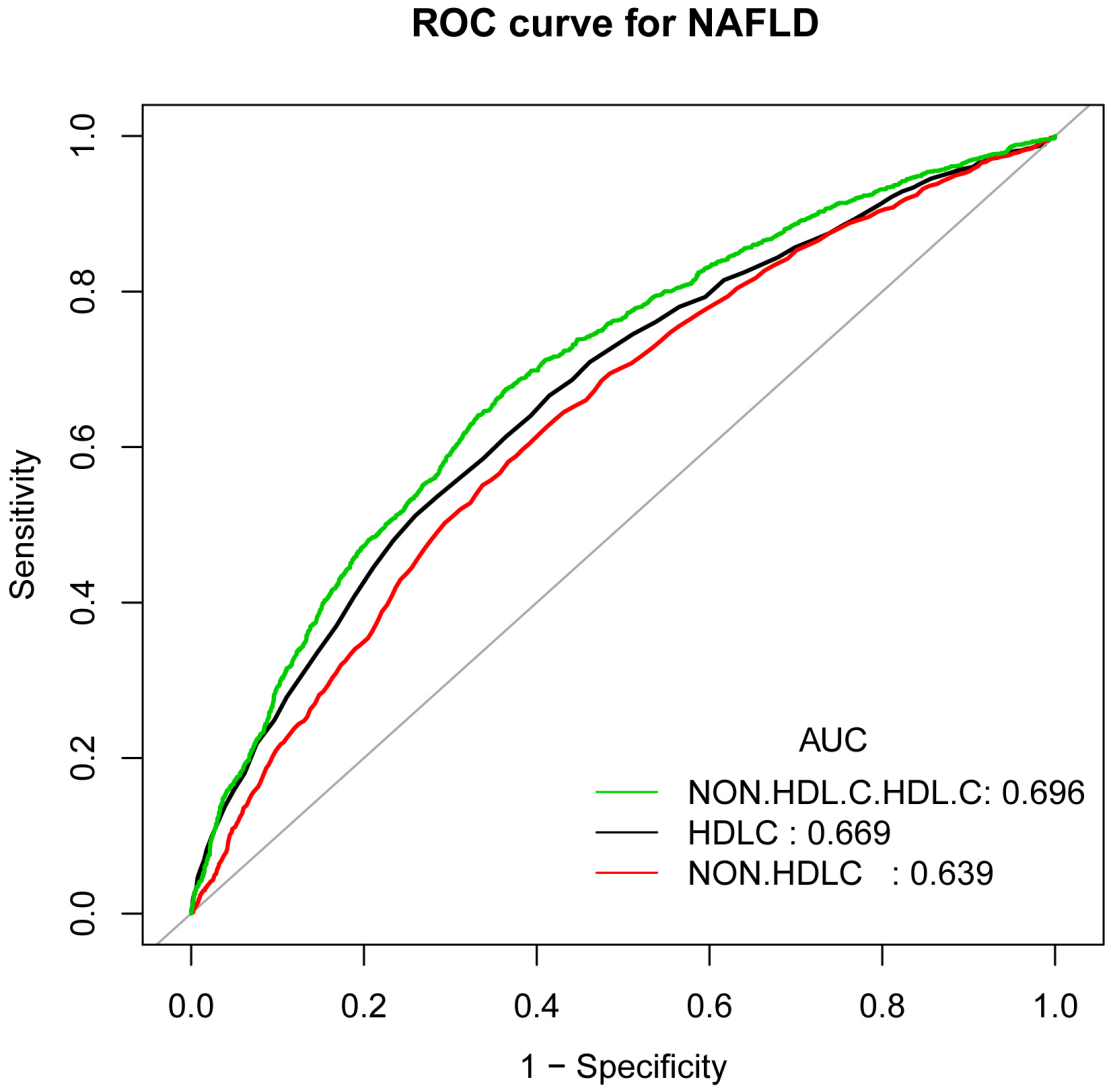
ROC curves for non-HDL-C/HDL-C, compared to non-HDL-C and HDL-C for NAFLD onset. As determined by AUC, the predictive value for non-HDL-C/HDL-C is more significant than other factors.

**Figure 4:**
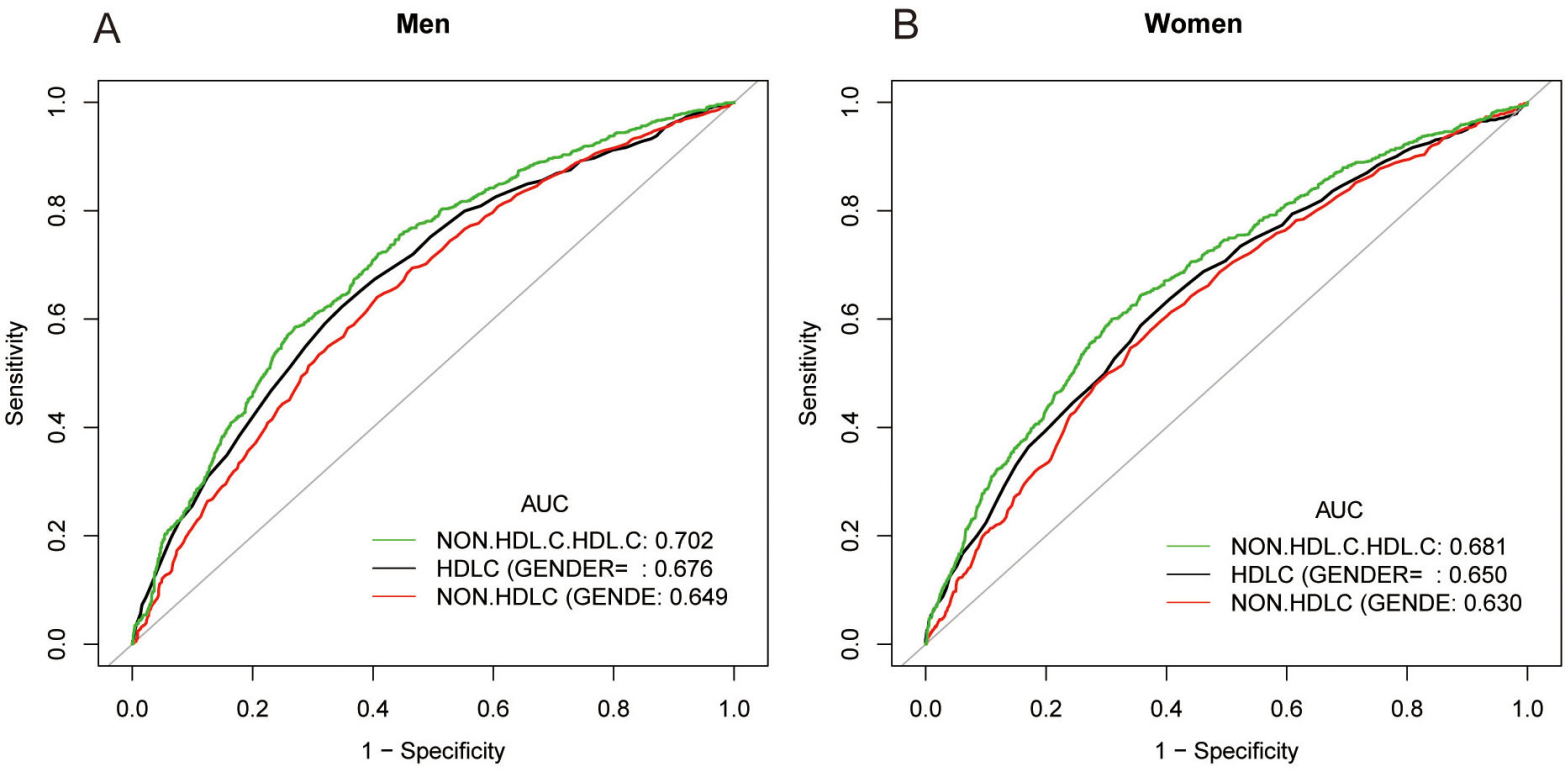
ROC curves for non-HDL-C/HDL-C, compared to non-HDL-C and HDL-C for NAFLD onset among males **(A)** and females **(B)**. As determined by AUC, the predictive value for non-HDL-C/HDL-C is more significant than those other factors.

In earlier studies using ROC curve analysis, HDL-C and non-HDL-C were significant predictors of NAFLD. **Figure 3** and **Supplementary Table 3** displayed the ROC for non-HDL-C/HDL-C compared to other lipid-related parameters. As shown in **Supplementary Table 3**, the ROC analysis’s area under the curve (AUC) for non-HDL-C/HDL-C was 0.6965 (95% CI: 0.6813, 0.7116), substantially more significant compared to that of HDL-C and non-HDL-C (*P* < 0.001). The estimated non-HDL-C/HDL-C’s sensitivity and specificity of NAFLD were 67.46% and 63.56%, respectively. Subgroup analysis based on gender was also carried out, and the results show that in the ROC analysis, the AUC for non-HDL-C/HDL-C was higher than the other indicators, as demonstrated in **Figure 4** and **Supplementary Table 2**.

## 8 Discussion

Using data from a nationwide cross-sectional study in the US, this paper primarily examined the relationships among non-HDL-C/HDL-C with NAFLD risk, hepatic steatosis severity, and hepatic fibrosis. In summary, the results mainly show that there may be a direct relationship between the elevated non-HDL-C/HDL-C/HDL-C ratio and the heightened risk of NAFLD and liver fibrosis in the general population. Interestingly, we found that each unit increase in non-HDL-C/HDL-C was linked to a 1.316-fold increase in the risk of NAFLD, even after correcting for all pertinent factors. Remarkably, significant positive relationships persisted in the subgroup analysis for both genders, particularly for men. Furthermore, we found a strong connection between non-HDL-C/HDL-C and the degree of hepatic steatosis. This study also revealed a strong association between non-HDL-C/HDL-C and significant liver fibrosis, advanced hepatic fibrosis, and cirrhosis in people, even after adjusting for all likely confounders. Furthermore, non-HDL-C/HDL-C was better at recognizing NAFLD than either non-HDL-C or HDL-C alone, according to the ROC analysis findings. More significantly, the non-HDL-C/HDL-C ratio is a relatively easy parameter to calculate, and this new measure might be a valuable tool for monitoring and controlling the risk of NAFLD and liver fibrosis in general participants.

According to several epidemiological and genetic research, dyslipidemia is a known pathogenic factor of NAFLD. Non-HDL-C is, as we all know, a general risk factor for cardiovascular disease. The association between non-HDL-C and the prevalence of NAFLD was initially observed by the authors in 2014; however, its link to the development of NAFLD has not been proven (9). Subsequent research has shown that an increased risk of NAFLD is associated with greater non-HDL-C levels (26, 27). In recent adult research, the capacity of non-HDL-C to differentiate between simple steatosis and NASH in NAFLD patients was examined (28). Non-HDL-C levels were considerably more significant in NASH patients than in individuals with simple steatosis when these patients did not use any lipid-lowering medication. According to the study’s findings, non-HDL-C can distinguish between steatosis and NASH using a noninvasive biomarker. The research has generally acknowledged the correlation between HDL-C and NAFLD status.

Furthermore, low HDL-C was a significant lipid anomaly strongly associated with the severity and progression of NAFLD (29, 30). Relevant studies by Alkassabany et al. demonstrate a substantial correlation between low HDL-C and high TG in school-age children with NAFLD (31). DeFilippis et al. discovered that in individuals of average weight, NAFLD is linked to decreased serum HDL-C (32). Obesity, insulin resistance, and dyslipidemia were also shown as significant risk factors for NAFLD in a recent study (32). Therefore, non-HDL-C and HDL-C combined have lately been proposed as a unique and more sensitive biomarker of inflammatory and metabolic illnesses.

So far, little research has examined the relationship between non-HDL-C/HDL-C and NAFLD. In 3374 Chinese people without liver illnesses or metabolic abnormalities, prospective cohort research revealed that the non-HDL-C/HDL-C ratio is an independent and stronger predictor of NAFLD than non-HDL-C (33). The research’s AUC was 0.682 for females and 0.717 for males. In addition, Gao et al. conducted a longitudinal study involving 16173 initially non-obese individuals without NAFLD who finished a 5-year follow-up investigation (34). The researchers found that the non-HDL-C/HDL-C ratio significantly predicted the cumulative incidence rate of NAFLD in non-obese individuals and that 2.26 was the threshold value for detecting NAFLD.

Similar to the prior study, the conclusion remains unchanged. Asian individuals, however, make up the bulk of the study subjects in these studies. The present research consistently shows a robust association between non-HDL-C/HDL-C and NAFLD. Additionally, we found that non-HDL-C/HDL-C had a significantly higher AUC for detecting NAFLD than other lipid markers. Our study showed that those with higher non-HDL-C/HDL-C had a greater chance of developing NAFLD than those with lower non-HDL-C/HDL-C. Significantly, our research also found a robust association between non-HDL-C/HDL-C and significant liver fibrosis, advanced liver fibrosis, and cirrhosis. We demonstrated that the non-HDL-C/HDL-C ratio is a valid marker of liver fibrosis for the first time. Our research further indicates the substantial positive correlation between non-HDL-C/HDL-C and the risk of NAFLD and liver fibrosis in a sizable general population in the United States. Although there are not many papers on this finding, we hope that more prospective, larger sample, multi-center and in-depth mechanistic studies will confirm it.

NAFLD is often accompanied by dyslipidemia, mainly represented by an increase in LDL-C and TG and a decrease in HDL-C. TC metabolism is closely related to the pathogenesis and severity of NAFLD. Free cholesterol acts on hepatic Kupffer cells and stele cells to produce inflammatory cytokines, thereby damaging liver cells and activating Kupffer cells to form an inflammatory circuit (35). Although the abnormal lipid metabolism in the early stage of NAFLD is the increase of TG content in the liver, TG itself is an inert lipid, and the deposition of ectopic TG may not have lipid toxicity (36). Many clinical and experimental data focus on regulating cholesterol homeostasis, pointing out that the disturbance of cholesterol homeostasis is a crucial metabolic factor in NAFLD lesions.

Free cholesterol can increase the liver’s sensitivity to inflammatory stimuli, and excessive accumulation of free cholesterol may lead to NASH (36). The impairment of cholesterol regulation may be a critical factor in NASH (37). Excess endogenous cholesterol can activate liver X receptors (LXRs), which regulate cholesterol homeostasis, induce liver lipopathy, and promote liver secretion of more VLDL particles (38, 39). LXRs also activate SREBP2, a nuclear transcription factor that regulates the expression of enzymes associated with cholesterol metabolism, as demonstrated by a 7-year follow-up prospective study that showed coding polymorphism can predict the occurrence and development of NAFLD (40). Excessive cholesterol intake activates this self-regulatory mechanism of the liver, which is likely to limit the lipid toxicity caused by excessive cholesterol accumulation by producing and exporting more non-HDL-C. However, this process may increase the production of arteriosclerotic lipoproteins, increasing the risk of cardiovascular disease in people with NAFLD.

Another recognized pathophysiology of NAFLD is IR. Reduced insulin sensitivity leads to a weakening of anti-lipolysis, which in turn leads to an increase in the concentration of plasma free fatty acid (FFA), an increase in FFA entering the liver, stimulation of liver synthesis, the release of very low-density lipoprotein (VLDL-C), and an increase in its hydrolyzed products. Lipoprotein lipase (LPL) is an enzyme dependent on insulin, and insulin stimulates the activity of LPL (41). Reduced insulin sensitivity was associated with lower LPL activity, delayed TG breakdown, TG-rich VLDL-C, and chylomicron (CM) catabolic blockage, which raised VLDL-C and CM blood levels. At the same time, due to the slow catabolism of VLDL and CM, the metabolites of ApoAl and phospholipid decrease, so the synthesis of new HDL-C is blocked, and the blood HDL decreases. The activity of LDL receptors decreases, and insulin regulates the binding of LDL-C and LDL-C receptors and the uptake of LDL-C. When insulin action reduces, LDL-C metabolism through the receptor pathway is blocked, and LDL-C in the serum increases (42, 43). Non-HDL-C includes LDL-C and VLDL-C, intermediate-density lipoprotein cholesterol (IDL-C), and other TG-rich lipoproteins, that is, the sum of various lipoprotein cholesterol except HDL-C, through the above mechanism eventually led to the increase of non-HDL-C in NAFLD. Therefore, the detection of non-HDL-C is helpful to comprehensively understand the lipid metabolism status of nonalcoholic fatty liver, including VLDL, IDL, and LP(a), and provides essential research parameters for exploring the pathophysiology of NAFLD itself.

We also offer further support for our investigation through gender-based stratified analysis. The subgroup analysis revealed that men with higher non-HDL-C/HDL-C levels were more likely to have NAFLD than women. Compared to women, men are more likely to have NAFLD (44, 45). This result may be related to the age of women in this study; the average age of women in the study is 43.35 ± 19.45, which was younger than menopausal age, and estrogen levels will be higher than menopause. Estrogen seems to play a vital role in liver lipid homeostasis. Estrogen produced by female ovaries can inhibit visceral fat accumulation, and the decreased estrogen level after menopause weakens the inhibitory effect on visceral fat deposition (46). Previous studies have shown that estrogen deficiency exacerbates NASH in a mouse model of fatty liver (45). Menopause’s lower estrogen levels may increase the chance of developing NAFLD. Compared to premenopausal women, postmenopausal women appear to have a greater prevalence of NAFLD. Therefore, we propose assessing the relevance of non-HDL-C/HDL-C in different sexes in predicting the risk of NAFLD.

The following are our study’s primary strengths. Firstly, the research’s most vital points are the large number of nationally representative populations and the generally accurate liver fibrosis and steatosis measurements. Secondly, this research is the first to show that the non-HDL-C/HDL-C ratio may independently predict liver fibrosis. There are a few limitations to the study. Firstly, the NHANES database constituted an observational cross-sectional study, inherently incapable of establishing a causal relationship between liver fibrosis, liver steatosis, and the ratio of non-HDL-C to HDL-C. Secondly, TE is used to diagnose NAFLD and liver fibrosis. Liver biopsy is the most reliable invasive technique to diagnose NAFLD and liver fibrosis. However, the current study used TE as a non-invasive technique without histological confirmation, which may have biased the results in the NAFLD patients. LSM assessment may be inaccurate in the presence of elevated serum ALT and may be overestimated in obese patients. Thirdly, the study population is predominantly non-Hispanic white/American. To address this issue, appropriate weighting was used in the data analysis to ensure that the results were representative of the US population in the statistical analysis. Future research should address these issues. However, non-HDL-C/HDL-C is easier to calculate and more widely available than other indices. It also shows a strong correlation with both hepatic fibrosis and hepatic steatosis.

## 9 Conclusion

Consequently, the present study’s findings revealed a significant independent association between elevated non-HDL-C/HDL-C and heightened risks of NAFLD and liver fibrosis within American cohorts. Utilizing non-HDL-C/HDL-C ratio holds promise as a predictive tool for both NAFLD and NAFLD-associated liver fibrosis.

## Data Availability

Publicly available datasets were analyzed in this study. This data can be found here: https://www.cdc.gov/nchs/nhanes/.
