# Supplementary material for: Elevated non-HDL-C to HDL-C ratio as a marker for NAFLD and liver fibrosis risk: a cross-sectional analysis
Source: Front Endocrinol (Lausanne). 2024 Oct 15;15:1457589. doi: 10.3389/fendo.2024.1457589 (PMC11518740; doi:10.3389/fendo.2024.1457589)
Supplement: Supplementary file 1 [file Table1.docx]

Supplementary Material

**1 Supplementary Figures and Tables**

- 1. **Supplementary Tables**

**Supplemental** **Table 1**. Correlation between non-HDL-C/HDL-C and hepatic steatosis based on CAP value.

|  | **Model 1 β (95% CI), *P* value** | **Model 2 β (95% CI), *P* value** | **Model 3 β (95% CI), *P* value** |
| --- | --- | --- | --- |
| **non-HDL-C/HDL-C** (per SD increase) | 16.924 (15.603, 18.245)  < 0.001 | 14.579 (13.267, 15.891)  < 0.001 | 5.638 (3.564, 7.713)  < 0.001 |
| Q1 (0.303-1.282) | Reference | Reference | Reference |
| Q2 (1.283-2.076) | 15.392 (10.661, 20.123) < 0.001 | 13.276 (8.714, 17.838)  < 0.001 | 3.838 (-2.671, 10.346) 0.247 |
| Q3 (2.077-3.204) | 40.801 (36.072, 45.529) < 0.001 | 34.702 (30.099, 39.305)  < 0.001 | 10.609 (3.971, 17.248)  0.001 |
| Q4 (3.205-9.951) | 61.120 (56.391, 65.849)  < 0.001 | 52.644 (47.941, 57.347)  < 0.001 | 14.180 (7.230, 21.131)  < 0.001 |
| *P* for trend | < 0.001 | < 0.001 | < 0.001 |
| **Subgroup analysis stratified by sex** | | | |
| Men | 14.425 (12.618, 16.233)  < 0.001 | 12.878 (11.123, 14.634)  < 0.001 | 5.674 (2.475, 8.872)  < 0.001 |
| Women | 19.756 (17.714, 21.797) < 0.001 | 17.150 (15.135, 19.165)  < 0.001 | 5.235 (2.428, 8.042)  < 0.001 |

Model 1: no covariates were adjusted. Model 2: age, gender, and race were adjusted. Model 3: age, gender, race, hypertension, statin use，waist circumference, hip circumference, BMI, T2DM, smoke, LSM, DBP, SBP, HBA1C, CRP, fast glucose, fast insulin, ALT, AST, GGT, TC, Physical activity, and SUA were adjusted. In the subgroup analysis for gender, the model was not adjusted for gender.

Abbreviations: OR Odds ratios, CI confidence interval,β standardization regression coefficient; other abbreviations as in **Table 1**.

**Supplemental** **Table 2**. Correlation between non-HDL-C/HDL-C and liver stiffness based on LSM value.

|  | **Model 1 β (95% CI), *P* value** | **Model 2 β (95% CI), *P* value** | **Model 3 β (95% CI), *P* value** |
| --- | --- | --- | --- |
| **non-HDL-C/HDL-C** (per SD increase) | 0.328 (0.223, 0.434)  < 0.001 | 0.308 (0.201, 0.415)  < 0.001 | 0.223 (0.096, 0.349)  < 0.001 |
| Q1 (0.303-1.282) | Reference | Reference | Reference |
| Q2 (1.283-2.075) | -0.081 (-0.457, 0.295)  0.67325 | -0.138 (-0.513, 0.237)  0.47055 | -0.098 (-0.552, 0.356) 0.67352 |
| Q3 (2.077-3.204) | 0.337 (-0.040, 0.714)  0.08002 | 0.225 (-0.153, 0.603)  0.24366 | 0.353(-0.100, 0.806)  0.12691 |
| Q4 (3.205-9.951) | 0.889 (0.508, 1.271)  < 0.001 | 0.780 (0.393, 1.166)  < 0.001 | 0.578 (0.113, 1.044)  0.01501 |
| *P* for trend | < 0.001 | < 0.001 | 0.00340 |
| **Subgroup analysis stratified by sex** | | | |
| Men | 0.321 (0.177, 0.466)  < 0.001 | 0.301 (0.154, 0.447)  < 0.001 | 0.295 (0.120, 0.471)  < 0.001 |
| Women | 0.338 (0.180, 0.497)  < 0.001 | 0.308 (0.147, 0.469)  < 0.001 | 0.137 (-0.054, 0.328)  0.15987 |

Model 1: no covariates were adjusted. Model 2: age, gender, and race were adjusted. Model 3: age, gender, race, hypertension, BMI, T2DM, smoke, CAP, DBP, SBP, HBA1C, CRP, fast glucose, fast insulin, ALT, AST, GGT, TC, Physical activity, and SUA were adjusted. In the subgroup analysis for gender, the model was not adjusted for gender.

Abbreviations: OR Odds ratios, CI confidence interval,β standardization regression coefficient; other abbreviations as in **Table 1**.

**Supplemental** **Table 3.** The best threshold, sensitivities, specificities, and area under the curve of lipid-related parameters for screening NAFLD in the general population and subgroup analysis for gender.

|  | **AUC** | **95% CI** | **Best threshold** | **Specificity** | **Sensitivity** |
| --- | --- | --- | --- | --- | --- |
| \| HDL-C \| 0.6686 \| 0.6530-0.6843 \| 46.5000 \| 0.7406 \| 0.5117 \| \| --- \| --- \| --- \| --- \| --- \| --- \| \| non-HDL-C \| 0.6388 \| 0.6229-0.6547 \| 124.5000 \| 0.5896 \| 0.6247 \| \| non-HDL-C/HDL-C \| 0.6965 \| 0.6813-0.7116 \| 2.4361 \| 0.6356 \| 0.6746 \| | | | | | |
| **Subgroup analysis stratified by sex**  **Men** | | | | | |
| HDL-C | 0.6761 | 0.6535-0.6986 | 45.5000 | 0.6515 | 0.6227 |
| non-HDL-C | 0.6493 | 0.6263-0.6723 | 123.5000 | 0.5930 | 0.6403 |
| non-HDL-C/HDL-C | 0.7021 | 0.6803-0.7239 | 2.9759 | 0.7295 | 0.5852 |
| **Women** |  |  |  |  |  |
| HDL-C | 0.6504 | 0.6284-0.6725 | 54.5000 | 0.5948 | 0.6367 |
| non-HDL-C | 0.6298 | 0.6075-0.6520 | 132.5000 | 0.6595 | 0.5466 |
| non-HDL-C/HDL-C | 0.6805 | 0.6591-0.7020 | 2.4356 | 0.6910 | 0.5998 |

Abbreviations: AUC area under the curve; other abbreviations are in **Table 1**.
